# 

**Published:** 1887-09

**Authors:** 


WHOLE NUMBER CCCXXVI. SEPTEMBER, 1887. NUMBER 2. VOLUME XXVIII.

### THE BUFFALO Medical and Surgical Journal

ESTABLISHED YEAR 1844.

LIBRARY INDEXED S.G.O.

**EDITORS:**

**THOS. LOTHROP, M. D.** **A. R. DAVIDSON, M. D.**

**ASSOCIATE EDITORS:**

FLOYD S. CREGO, M. D. CARLTON C. FREDERICK, M. D.
FRED. R. CAMPBELL, M. D. FRANK H. POTTER, M. D.

EDWARD B. ANGELL, M. D., Rochester. N. Y.

